# Expression of miR-127 and miR-29 in Egyptian patients with Behçet disease and its clinical significance and relationship with disease activity

**DOI:** 10.1007/s10067-025-07470-4

**Published:** 2025-05-13

**Authors:** Heba E. Tolba, Yasser E. Taha, Nermeen A. Fouad, Omayma O. Abdelaleem

**Affiliations:** 1 Department of Rheumatology and Rehabilitation, Faculty of Medicine, Fayoum University, Fayoum, Egypt; 2 Department of Medical Biochemistry and Molecular Biology, Faculty of Medicine, Fayoum University, Fayoum, Egypt

**Keywords:** Behçet disease, MiR-29, MiR-127

## Abstract

**Background:**

Behçet’s disease (BD) is characterized by a variety of clinical involvements and unpredictable courses of remission and exacerbation. BD cannot be diagnosed based on any particular laboratory, histopathologic, or genetic data. After excluding other possible causes, the diagnosis of BD is mostly made based on clinical manifestations. This research aims to assess the expression of miR-127 and miR-29 in Behçet patients and to correlate this expression with different disease manifestations and disease activity.

**Methods:**

Seventy adult patients with Behçet disease and 30 matched controls were enlisted. Behçet’s Disease Current Activity Form (BDCAF) was used to determine the patients’ activity scores. Gene expressions of miR-127 and miR-29 were assessed using real-time PCR.

**Results:**

miR-29 was statistically significantly higher in cases when compared to controls with *p* < 0.001. Patients with oral ulcers, genital ulcers, and neurological manifestations had a significantly higher levels of miR-29b (*p* = 0.005, 0.008, 0.003, respectively). Expression level of miR-29b was higher in patients with active disease compared with those who were in inactive state (*p* = 0.003). Patients with high and moderate severity score had a statistically significant higher expression level of miR-29 when compared to patients with mild severity score. There was a statistically significant positive correlation between miR-29 and several study parameters including BDCAF (*r* = 0.440, *p* < 0.001) and severity score (*r* = 0.243, *p* = 0.043). miR-127 was statistically significantly lower in cases when compared to controls with *p* < 0.001. There were statistically significant negative correlations between miR-127 and each of ESR (*r* = − 0.361, *p* = 0.002) and BDCAF (*r* = − 0.350, *p* = 0.003).

**Conclusions:**

miR-29 expression level was statistically significantly higher while miR-127 was statistically significantly lower in patients with Behçet disease compared with control individuals. Furthermore, miR-29 was significantly positively correlated whereas miR-127 was significantly negatively correlated with BDCAF denoting their association with disease activity.

**Table:** 

**Key Points** • *Behçet’s disease (BD) is a systemic inflammatory disease with uncertain etiology. There is currently no diagnostic test for Behçet disease. Several miRNAs have been identified as powerful diagnostic biomarkers in many diseases*. • *Measuring the expression levels of miR-29 and miR-127 in serum of sample of patients with Behçet disease was performed in the current study*. • *miR-29 expression level was statistically significantly higher while miR-127 was statistically significantly lower in patients with Behçet disease compared with control individuals. Furthermore, miR-29 was significantly positively correlated whereas miR-127 was significantly negatively correlated with BDCAF denoting their association with disease activity*.

## Introduction

Behçet's disease (BD) is a type of variable vessel vasculitis that affects multiple organs and systems. It can lead to various skin lesions such as papules and pustules, ulcers on the oral, genital, and intestinal mucosa, arthritis, uveitis, lesions in the central nervous system, venous and arterial thrombosis, and arterial aneurysms [1]. A single-center study from Egypt revealed a male/female ratio of 5.4:1 and a prevalence of 7.6/100,000 people [2]. Currently, there is no diagnostic test for Behçet disease and patients with active disease may have normal levels of inflammatory markers such C-reactive protein [3].

It has been revealed that peripheral blood mononuclear cells (PBMCs) from individuals with BD exhibit disrupted microribonucleic acid (miRNA) expression, and several miRNAs have been identified as powerful diagnostic biomarkers in addition to their roles in pathogenesis of the diseases [4]. The miR-29 family, which is expressed in both T and B cells was found to play a function in Toll-like receptor inhibition, promote DNA demethylation, and activate the AKT signaling pathway [5]. miR-29 targets IFN-γ and NF-κB genes which promote chronic inflammation and documented to be increased in Behçet disease [6, 7]. miR-29 induces inflammation by triggering the NF-κB and Janus kinase/signal transducers and activators of transcription (JAK/STAT) signaling pathways in endothelial cells [7].

The two mature miRNAs, miR-127-3p and miR-127-5p, are derived from the same precursor miRNA; henceforth, miR-127-3p shall be denoted as miR-127. miR-127 regulates inflammatory cell infiltration and affects the production of inflammatory cytokines. IL-6, TNF-α, and IL-1β are examples of inflammatory cytokines that can promote the synthesis of other cytokines and effector molecules that can aid in the treatment of a specific illness [8]. miR-127-3p has been demonstrated to function as a negative regulator of the type I interferon (IFN-I) signaling pathway by preventing the phosphorylation of STAT proteins and the stimulation of gene expression triggered by IFN-α through the interferon stimulated response element (ISRE) or GAS [9]. Assessing the expression of two miRNAs (miR-127 and miR-29) in Behçet patients and correlating this expression with various illness manifestations and disease activity was the aim of the current study.

## Patients and methods

Seventy adult BD patients who met the international criteria for BD (ICBD) [10] were gathered from the outpatient clinic and inpatient wards of the rheumatology department. Individuals suffering from additional autoimmune conditions including juvenile BD, chronic diseases, people using drugs (statins, angiotensin-converting enzyme inhibitors) that affect endothelial cells, smokers, or those with history of infection (< 3 months) were excluded from the study. Thirty age- and sex-matched healthy controls were also included in the study**.** At baseline, all patients and control subjects gave their informed consent to take part in the study, and the Ethics Committee of Biomedical Research approved it (M 626).

The Behçet’s Disease Current Activity Form (BDCAF) [11] and Behçet severity score [12] were assessed.

Sera collected from each participant were utilized for microRNA extraction and real-time PCR to determine fold changes of miR-127 and miR-29b.

The detailed methodology was mentioned in supplementary file.

### Statistical method

Software called the Statistical Package for the Social Sciences (SPSS version 22) was used to analyze the data. Qualitative data were presented as numbers and percentages; the differences were evaluated using chi squared test. Quantitative variables were expressed as mean and standard deviation (SD); independent *t* test was performed in comparing the two study groups. Study markers were presented as median and interquartile range (IQR); Mann–Whitney *U* test or Kruskal–Wallis test were utilized in comparison between two or three groups, respectively. Spearman correlation was run to test the relation between study markers and the quantitative variables. Receiver operating characteristic (ROC) curve analysis was done to evaluate the discriminative power of miR-127 and miR-29 to differentiate cases from control. A statistically significant *p* value was defined as less than 0.05.

## Results

### Demographic characteristics of patients with Behçet disease and controls

The mean age of the cases was 39.6 ± 10.4 years while for controls was 38.1 ± 12.5 (*p* = 0.558). No significant differences between the cases and the controls regarding sex (*p* = 0.736). Clinical manifestations, disease activity, and severity are shown in Tables 1 and 2.

**Table 1:** Clinical manifestations of patients with Behçet disease

		Number	Percent
Oral ulcers	Positive	58	82.9
Genital ulcers	Positive	42	60.0
Cutaneous lesion	Positive	40	57.1
Ocular manifestations	Positive	47	67.1
Vascular manifestations	Positive	17	24.3
Neurological manifestation	Positive	14	20.0
Musculoskeletal manifestations	Positive	56	80.0
Pathergy test	Positive	29	41.4

**Table 2:** Disease activity and severity of Behçet patients under the study

BDCAF	Mean ± SD	2.6 ± 1.9	
Severity score	Mean ± SD	6.0 ± 1.8	
		*N*	%
Activity (BDCAF)	Active	43	61.4
	Inactive	27	38.6
Behcet severity score	Mild	8	11.4
	Moderate	33	47.1
	High	29	41.4

*BDCAF* Behçet Disease Current Activity Form

### Serum expression levels of miR-29 and miR-127 in patients with Behçet disease and controls and their relations with various clinical and laboratory data

**Fig. 1:**
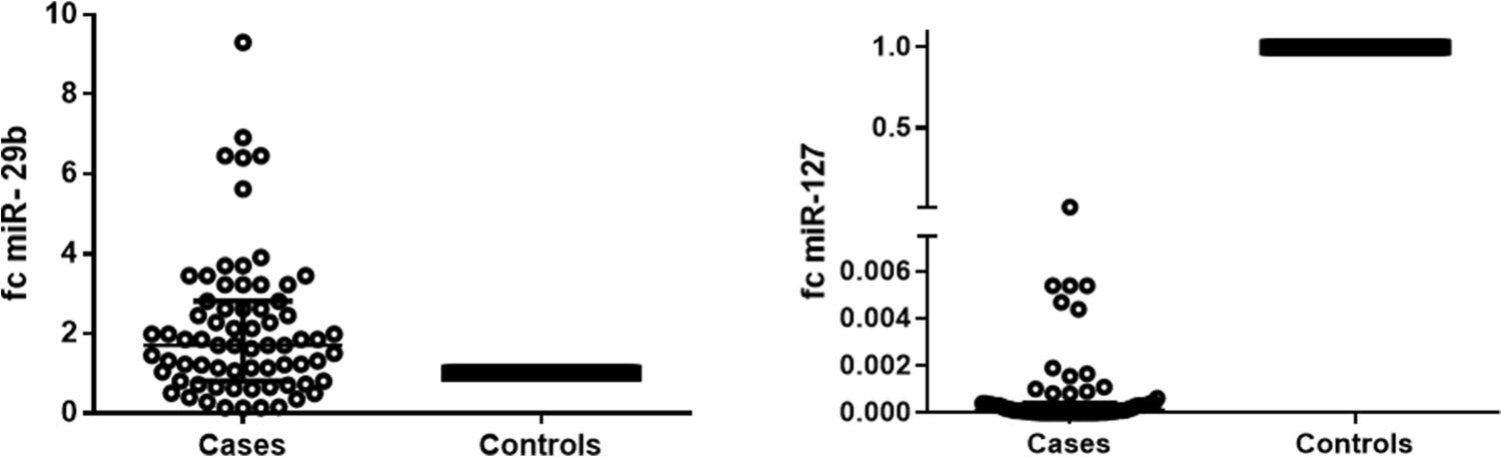
Differences in serum miR-29 and miR-127 between patients with Behçet disease and controls. miR-29 was a statistically significantly higher in cases when compared to controls (median (range) = 1.706 (0.807–2.809) with *p* < 0.001. miR-127 was a statistically significantly lower in cases when compared to controls (median (range) = 0.00011 (0.00001–0.00041) with *p* < 0.001. Horizontal dotted line indicated the expression levels of miR-29 and miR-127 in controls (since 2^0^ = 1 and − ΔΔCt for controls equals 0, the control value was set to 1)

miR-29 was a statistically significantly higher in cases when compared to controls [median (range) = 1.706 (0.807–2.809)] with *p* < 0.001 as shown in Fig. 1. Patients with oral ulcers, genital ulcers, and neurological manifestations had a significantly higher levels of miR-29b (*p* = 0.005, 0.008, 0.003, respectively). Expression level of miR-29b was higher in patients with active disease (*p* = 0.003). Patients with high and moderate severity score had a statistically significant higher expression level of miR-29 when compared to patients with mild severity score Table 3. There was a statistically significant positive correlation between miR-29 and several study parameters including BDCAF (*r* = 0.440, *p* < 0.001) and severity score (*r* = 0.243, *p* = 0.043) (Table 5). On the other hand, miR-127 was a statistically significantly lower in cases when compared to controls [median (range) = 0.00011 (0.00001–0.00041)] with *p* < 0.001 (Fig. 1). There was no statistical difference in the expression level of miR-127 as regard other disease characteristics as demonstrated in Table 4. There were statistically significant negative correlations with ESR (*r* = − 0.361, *p* = 0.002) and BDCAF (*r* = − 0.350, *p* = 0.003) (Table 5).

**Table 3:** Relation between miR-29 and characteristics of Behçet patients

		miR-29			*p* value
		Median	IQR		
Oral ulcers	Negative	0.717	0.447	1.009	0.005*
	Positive	1.853	1.223	2.809	
Genital ulcers	Negative	1.176	0.552	1.991	0.008*
	Positive	1.986	1.31	3.227	
Activity	Active	2.282	1.706	3.227	0.003*
	Inactive	1.227	0.621	2.129	
Severity	Mild	0.657	0.447	0.807	0.012* 0.003* 1.000
	Moderate	1.853	1.227	3.227	
	Severe	1.986	1.041	2.809	
Cutaneous lesion	Negative	0.717	0.447	1.009	0.182
	Positive	1.853	1.223	2.809	
Ocular manifestations	Negative	1.176	0.552	1.991	0.107
	Positive	1.986	1.31	3.227	
Vascular manifestations	Negative	1.92	1.141	3.458	0.607
	Positive	1.562	0.681	2.537	
Neurological manifestation	Negative	1.227	0.497	2.621	0.003*
	Positive	1.853	1.141	2.809	
Joints	Negative	1.706	0.807	2.809	0.901
	Positive	1.986	1.141	2.282	
Pathergy test	Negative	1.562	0.712	2.367	0.294
	Positive	3.227	1.986	3.706	
	Positive	1.706	0.727	3.227	
	Positive	1.706	0.767	2.715	

*Significant

**Table 4:** miR-127 and different characteristics of Behçet patient

		miR-127			*p* value
		Median	IQR		
Oral ulcers	Negative	0.00001	0.00001	0.0001	0.009*
	Positive	0.00022	0.00002	0.00063	
Genital ulcers	Negative	0.0001	0.00001	0.00036	0.569
	Positive	0.00025	0.00001	0.00042	
Activity	Active	0.00022	0.00001	0.00042	0.526
	Inactive	0.0001	0.00001	0.00036	
Severity	Mild	0.00005	0.00001	0.00026	0.228
	Moderate	0.00007	0.00001	0.00037	
	Severe	0.00027	0.00006	0.00063	
Cutaneous lesion	Negative	0.00008	0.00001	0.00082	0.700
	Positive	0.0002	0.00002	0.00037	
Ocular manifestations	Negative	0.00008	0.00001	0.00036	0.507
	Positive	0.00017	0.00001	0.00082	
Vascular manifestations	Negative	0.0001	0.00001	0.00041	0.373
	Positive	0.00022	0.00006	0.00041	
Neurological manifestation	Negative	0.0001	0.00001	0.00052	0.719
	Positive	0.0002	0.00006	0.00036	
Musculoskeletal	Negative	0.00015	0.00002	0.00041	0.809
	Positive	0.0001	0.00001	0.00041	
Pathergy test	Negative	0.0001	0.00001	0.00063	0.919
	Positive	0.00017	0.00002	0.00032	

*Significant

**Table 5:** Correlations of miR-29 and miR-127 with different patient and laboratory analyses

		miR-29	miR-127
HB	*r*	0.074	0.123
	*p* value	0.541	0.311
TLC	*r*	0.152	0.108
	*p* value	0.210	0.372
Platelets	*r*	− 0.105	− 0.039
	*p* value	0.386	0.751
ESR	*r*	0.039	− 0.361
	*p* value	0.751	0.002*
CRP	*r*	0.044	− 0.154
	*p* value	0.719	0.205
Urea	*r*	0.029	0.043
	*p* value	0.811	0.723
Creatinine	*r*	− 0.108	0.045
	*p* value	0.372	0.714
ALT	*r*	− 0.074	− 0.074
	*p* value	0.542	0.544
AST	*r*	− 0.005	0.125
	*p* value	0.967	0.308
RBS	*r*	0.064	− 0.190
	*p* value	0.597	0.115
BDCAF	*r*	0.440	− 0.350
	*p* value	< 0.001*	0.003*
Severity score	*r*	0.243	0.276
	*p* value	0.043*	0.021*

*HB* hemoglobin, *TLC* total leukocyte count, *ESR* erythrocyte sedimentation rate, *CRP* C-reactive protein, *ALT* alanine transaminase, *AST* aspartate aminotransferase, *RBS* random blood sugar, *BDCAF* Behçet Disease Current Activity Form

*Significant

### ROC analysis discriminating patients with Behçet disease from control subjects using expression levels of miR-29 and miR-127

**Fig. 2:**
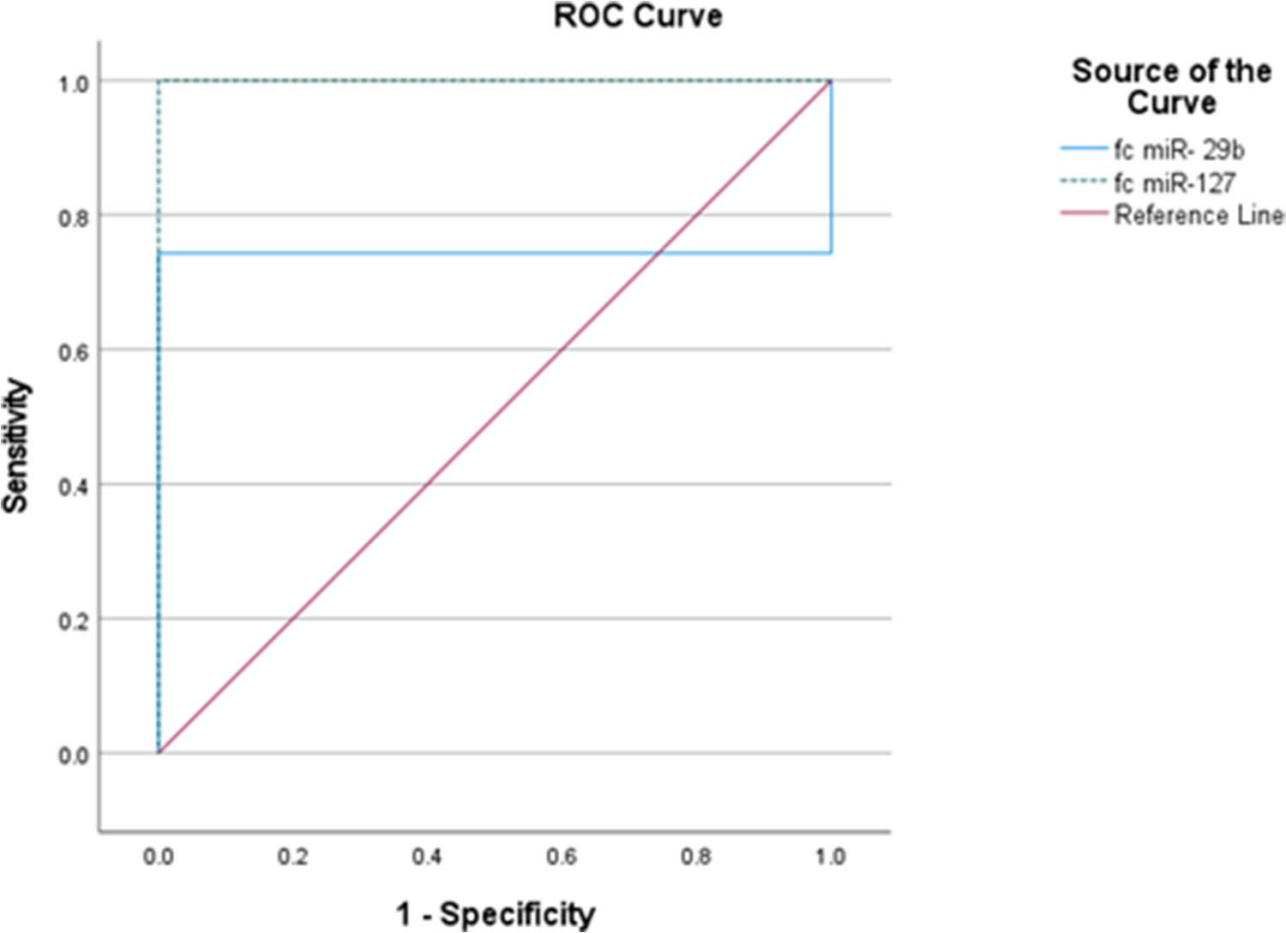
ROC curve analysis for miR-127 and miR-29

miR-29 had an excellent discriminative power for differentiating Behçet cases from controls [AUC = 0.743, 95% CI = (0.640–0.845)] with sensitivity of 74.3% and specificity of 100% (*p* < 0.001). Also, miR-127 had an excellent discriminative power for differentiating Behçet cases from controls [AUC = 1.00, 95% CI = (1.00–1.00)] with sensitivity of 100% and specificity of 100% (*p* < 0.001) as observed in Fig. 2.

## Discussion

miRNA dysregulation in BD patients may lead to aberrant amounts of immune-suppressive and inflammatory cells and cytokines [10]. This study was designed to assess expression of miR-29 and miR-127 in BD patients and to correlate this expression with different disease manifestations and disease activity. The expression levels of miR-29 were significantly upregulated in blood samples of BD patients when compared with controls (*p* ≤ 0.001); up to our knowledge, this is the first study which assesses miR-29. miR-29 enhances lipopolysaccharide-induced inflammation by activating the NF-κB and JAK/STAT signaling pathways in endothelial cells [7]. Additionally, mature B lymphocytes in the periphery were found to activate PI3 K signaling through the miR-29 family of miRNAs [11]. The complicated pathophysiology of BD includes the activation of several signaling pathways, such as the nuclear factor kappa B (NF-κB) signaling system and the phosphatidylinositol 3-kinase (PI3 K)/protein kinase B (AKT) signaling network [10]. So, this may explain the elevation of miR-29 in patients with Behçet disease.

Current findings are in line with other studies that were highlighting higher levels of miR-29 in patients with rheumatoid arthritis [12] and systemic lupus erythematosus (SLE) [5], suggesting that it may be considered as a biomarker for their susceptibility. Similarly, another study verified that miR-29 was upregulated in cartilage tissue from patients with OA when compared to controls. miR-29 could significantly downregulate progranulin expression which play an important role in cartilage formation and function and reported to have anti-inflammatory functions [13]. Furthermore, a previous study found that miR-29 was increased in pediatric Crohn’s disease [14]. In contrast, a former study found that systemic sclerosis fibroblasts and skin sections had much lower levels of miR-29a than healthy control samples [15].

The current study found that miR-29 was statistically significantly higher in patients with active disease (*p* = 0.003), and there was a statistically significant positive correlation between miR-29 and BDCAF (*p* < 0.001) and severity score (*p* = 0.043). Patients with high and moderate severity score had a statistically significant higher expression level of miR-29 when compared to patients with mild severity score. With review to literatures, a previous study found that miR-29b expression was upregulated in SLE and correlated with SLEDAI score, anti-dsDNA, and complement C3 level in patients with SLE [5]. Similarly, a study done by Ren et al. reported that upregulation of miR-29b was correlated with RA disease activity [12]. This study found that miR-29 had an excellent discriminative power for differentiating Behçet cases from controls (AUC = 0.743, 95% CI = (0.640–0.845). The optimal cutoff point was 1.02, at which sensitivity was 74.3% and specificity was 100.0%. These results are in line with a study of SLE done by Wang et al., who found that miR-29 had area under the curve 0.752, denoting a good diagnostic power for SLE (AUC > 0.75).

As regard to miR-127, the current study found that miR-127 expression level was statistically significantly lower in cases when compared to controls *p* < 0.001. Our study is the first to study the role of miR-127 in BD disease. A previous study done by Wu et al. proved that miR-127 expression level was downregulated in the kidney of lupus nephritis [16]. Downregulation of miR-127 promotes Janus family of kinases (JAK1) expression and contributes to the abnormal activation of IFN-I signaling pathway in the kidney of LN [16]. There is some similarity between SLE and Behçet as JAK1/STAT3 signaling pathway is activated in BD, possibly through elevated serum and tissue expressions of Th1/Th17 type cytokines [17]. Another previous study done by Umeh-Garcia et al. found that expression level of miR-127 was reduced in breast cancer through targeting the PI3 K/Akt pathway [18]. Our current study found that miR-127 had an excellent discriminative power for differentiating Behçet cases from controls [AUC = 1.00, 95% CI = (1.00–1.00)]. The optimal cutoff point for 0.51 at which sensitivity was 100% and specificity was 100.0%.

A small number of participants who shared the same ethnic background participated in the current study is a limitation of our study; therefore, a more comprehensive investigation encompassing multiple countries might enhance the results.

In conclusion, miR-29 was a statistically significantly higher in patients with BD. There was a statistically significant positive correlation between miR-29 and BDCAF, expression level of miR-29 was higher in patients with active disease. On the other hand, miR-127 was a statistically significantly lower in patients with Behçet disease, miR-127 was negatively correlated with BDCAF. miR-29 and miR-127 could be considered as useful diagnostic markers for differentiating patients with BD from controls.

## Data Availability

The datasets used and/or analyzed during the current study available from the corresponding author on reasonable request.

## Abbreviations

BD: Behçet disease
BDCAF: Behçet Disease Current Activity Form
miRNA: MicroRNA
ISRE: Interferon stimulated response element
ISGs: Induction of IFN stimulated genes
STAT: Signal transducer and activator of transcription
ICBD: International Criteria for Behçet Disease
FC: Fold change
SD: Standard deviation
